# Iatrogenic Aortocoronary Dissection: Management and Outcomes

**DOI:** 10.1016/j.jaccas.2025.104888

**Published:** 2025-09-03

**Authors:** Ravi Sankar Tulluru, Karthik Gopinath, John Jose E, Viji Samuel Thomson, Ommen K. George

**Affiliations:** Department of Cardiology, Christian Medical College and Hospital, Vellore, India

**Keywords:** coronary angiogram, coronary dissection, CT aortogram, ostial stenting, percutaneous coronary intervention

## Abstract

**Background:**

Iatrogenic aortocoronary dissection (IACD) is a rare but potentially life-threatening complication of percutaneous coronary intervention or diagnostic angiography. The increasing complexity of interventions, especially cases involving chronic total occlusions, calcified lesions, and aggressive balloon dilation, has heightened the risk of IACD. It is often underreported, with an estimated incidence of 0.02% to 0.10%.

**Case Presentation:**

We present 4 patients with IACD who were managed at our institute in 2024. The right coronary artery was the most frequently involved vessel, and type III IACD was the most common variant. Management strategies included stenting for 50% of patients and conservative management for 50%; no patient required surgical intervention.

**Conclusions:**

All patients remained stable and were doing well at follow-up. Our case series provides valuable insights into the risk factors, clinical presentation, and management of IACD, highlighting the importance of prevention, early recognition, and tailored treatment strategies to optimize patient outcomes.

## Visual Summary

**Figure:**
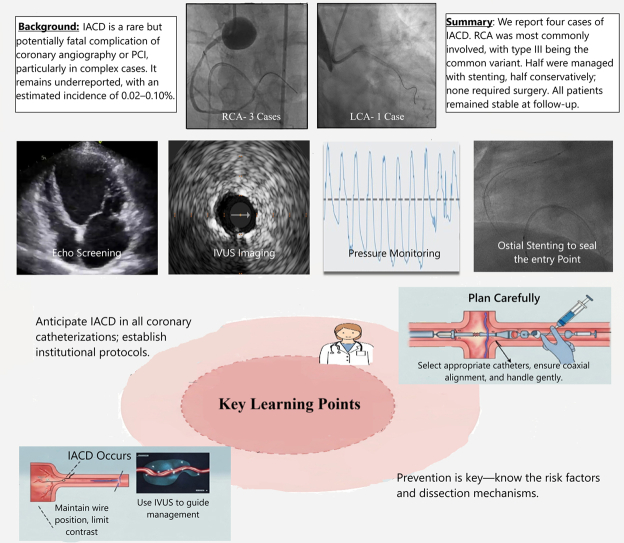

**Figure 1:**
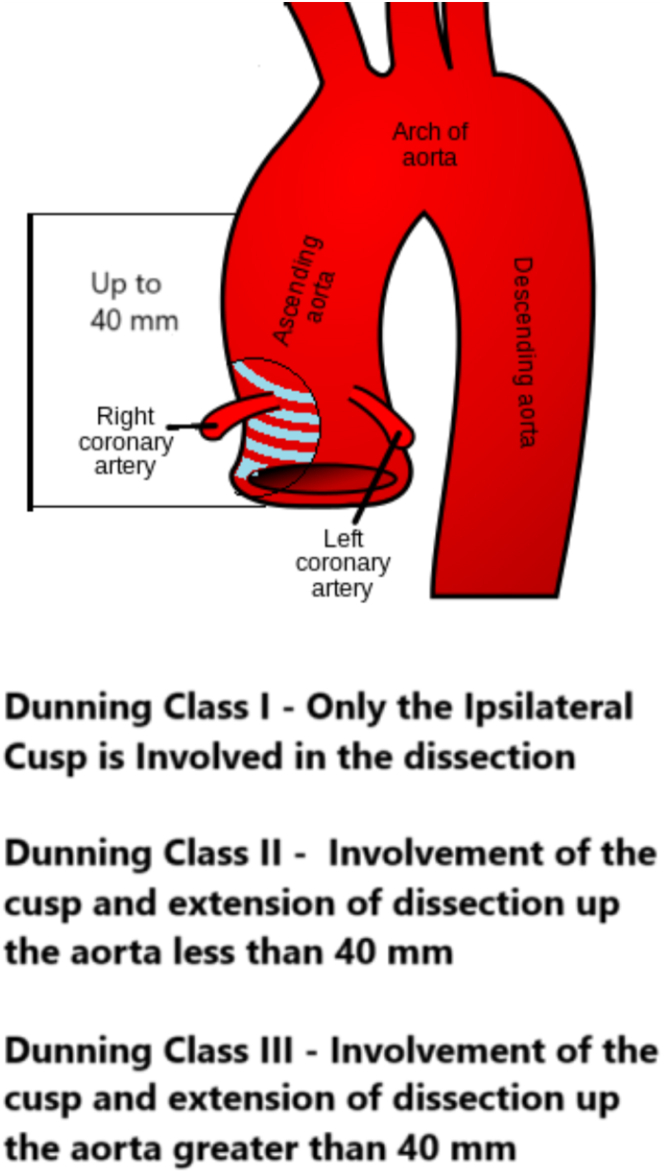
Aortocoronary Dissection Classification Proposed by Dunning et al^2^ in 2000 Diagram showing the 3 types of IACD as classified by Dunning et al.^2^

Iatrogenic aortocoronary dissection (IACD) is a rare but potentially fatal complication of percutaneous coronary intervention (PCI) or diagnostic angiography. IACD arises from unintentional intimal damage of the coronary artery or aortic root due to catheter manipulation or inadvertent contrast injection. Prognosis depends on the extent of aortic involvement, aortic valve complications, and coronary perfusion. Clinical presentations range from asymptomatic angiographic findings to severe myocardial ischemia, arrhythmias, cardiogenic shock, or even death.^1^ Dunning et al^2^ classified IACD into 3 types based on the extent of aortic involvement (Figure 1).

**Take-Home Messages**

• Consider IACD as a potential complication in all coronary procedures and establish clear management protocols, focusing on prevention through understanding of risk factors and dissection mechanisms.
• Conduct thorough preprocedural assessment, choose appropriate catheters, ensure coaxial alignment, and handle catheters and wires gently, especially in high-risk cases.
• If dissection occurs, maintain wire access, minimize contrast use, and utilize intravascular ultrasound for precise management.

The rising prevalence of PCIs in chronic total occlusions (CTOs), calcified lesions, and aggressive balloon dilations have markedly elevated the risk of IACD. It is often underreported, with an estimated incidence of 0.02% to 0.10% and a mortality rate between 5% and 10%.^3^, ^4^, ^5^ Our institute performs roughly 5,000 coronary procedures annually, with IACD occurring in 1 to 4 patients. We describe 4 such patients from 2024 to highlight management approaches and preventive strategies (Table 1).

**Table 1:** Comprehensive Patient Review and Analysis

	Patient 1	Patient 2	Patient 3	Patient 4
Age/sex	45/female	74/male	65/female	51/male
Risk factors	Diabetes, hypertension	Diabetes, hypertension	None	Smoker
Prior PCI/MI/CVA	None	None	Inferior wall MI, PCI to RCA 3 d before	PCI to RCA 6 y before
Ejection fraction (%)	54	38	42	42
Presentation	MI	SIHD	Post-MI	SIHD
Culprit vessel	RCA	RCA	LCA	RCA
Catheter/access	6-F Judkins right 3.5 / femoral	6-F Judkins right 3.5 / radial	6-F extra-backup LAD 3.0 / radial	7-F Amplatz left 1 / femoral
IACD type (Dunning)	III	III	III	II
Possible mechanism of dissection	Deep catheter engagement + contrast injection into the ballooned segment	Excessive postdilation of the ostial RCA stent	Deep catheter engagement + wedged contrast injection	Deep catheter engagement + contrast injection into the ballooned segment
Management strategy	Ostial stenting	Conservative (wait and watch)	Ostial stenting	Conservative (wait and watch)
Intravascular imaging	IVUS	None	None	None
Post-PCI imaging	Echocardiography daily + CT scan (day 3)	Echocardiography daily + CT scan post-PCI	Echocardiography alone	Echocardiography daily + cardiac MRI (1 mo)
Outcome	Stable; discharged after 5 d	Stable; discharged after 5 d	Stable; discharged after 3 d	Stable; discharged after 3 d
Follow-up	Doing well for 1 y	Doing well for 3 mo	Doing well for 2 mo	Doing well for 6 mo

CT = computed tomography; CVA = cerebrovascular accident; IACD = iatrogenic aortocoronary dissection; IVUS = intravascular ultrasound; LAD = left anterior descending artery; LCA = left coronary artery; MI = myocardial infarction; MRI = magnetic resonance imaging; PCI = percutaneous coronary intervention; RCA = right coronary artery; SIHD = stable ischemic heart disease.

## Patient 1

**Figure 2:**
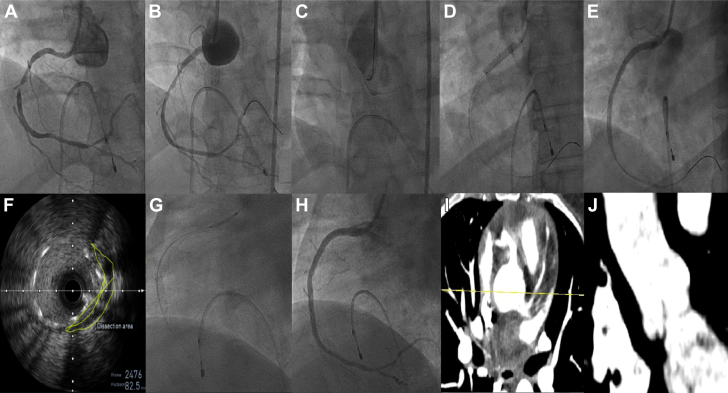
Angiographic, Ultrasonographic, and CT Images From Patient 1 Demonstrating Progression and Management of IACD (A) Angiogram showing a tight lesion in the mid-RCA. (B) Type III IACD postballooning. (C) Disengaged Judkins right catheter postevent. (D) Stenting of mid to proximal RCA after successful rewiring. (E) Persistent contrast extravasation poststenting. (F) IVUS of RCA ostium showing incomplete coverage. (G) Additional stenting extended into the aortic sinus. (H) Final angiogram showing sealed-off dissection with good distal flow. (I and J) CT aortogram (transverse and coronal) confirming sealed-off aortic dissection without further propagation. CT = computed tomography; IACD = iatrogenic aortocoronary dissection; IVUS = intravascular ultrasound; RCA = right coronary artery.

A 45-year-old woman presented with an inferior wall myocardial infarction at a 4-hour window period. Angiogram showed double-vessel coronary artery disease with a recanalized mid–right coronary artery (RCA). The RCA was dilated after engaging with a Judkins right catheter. Postdilation, contrast injection into the ballooned segment caused a Dunning type III IACD extending from the RCA ostium into the ascending aorta. During catheter disengagement, the guide wire accidentally slipped out. The patient subsequently experienced acute chest pain with hypotension, and echocardiography revealed a dissection flap with mild aortic regurgitation. Given ongoing symptoms, ostial stenting was preferred over surgery. Inotropes were increased to maintain adequate mean arterial pressure. The mid to proximal RCA was stented with 2 drug-eluting stents after successful wiring with partial engagement of the catheter (using the no-touch technique) and confirming the true lumen with intravascular ultrasound. The check angiogram revealed persisting extravasation, and intravascular ultrasound showed incomplete coverage in 1 quadrant, necessitating an additional stent. Final angiogram demonstrated TIMI flow grade 3 without extravasation. A computed tomography (CT) aortogram after 2 days confirmed sealed-off aortic dissection (Figure 2, Video 1). The patient was doing well at the 1-year follow-up. This event might have been prevented by avoiding wedged injection into the diseased segment and ensuring coaxial catheter alignment.

## Patient 2

**Figure 3:**
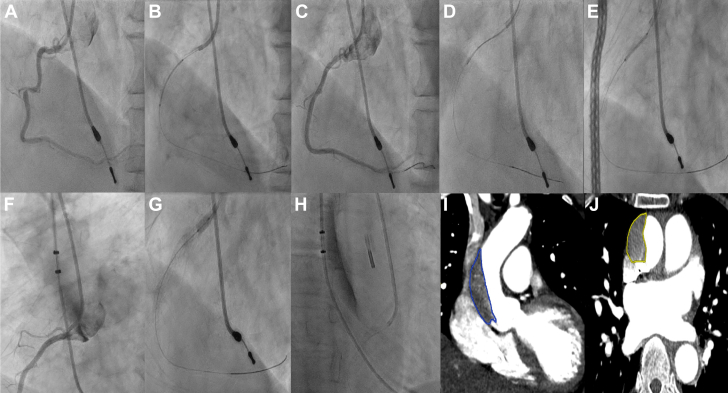
Angiographic and CT Images From Patient 2 Illustrating Development and Conservative Management of IACD (A) Angiogram showing a tight mid-RCA lesion. (B) Stenting of the mid-RCA. (C) Poststenting angiogram showing good result with minor ostial disease. (D) IVUS assessment revealing a significant ostial RCA lesion. (E) Extension of stenting to the RCA ostium. (F) Type III IACD after proximal stent postdilation. (G) Intermittent balloon occlusion of the RCA stent. (H) Persistent dissection without progression after 30 minutes. (I and J) CT aortogram (coronal and transverse) showing false lumen thrombosis. Abbreviations as in Figure 2.

A 74-year-old man with distal left main plus double-vessel coronary artery disease was admitted for elective angioplasty. The RCA was engaged with a Judkins right catheter and was stented with 3-mm and 3.5-mm drug-eluting stents from the ostium, then postdilated using 3.5-mm and 4-mm noncompliant balloons at 10 to 16 atm after intravascular ultrasound assessment. This resulted in a Dunning type III IACD with TIMI flow grade 3 down the vessel. Echocardiography showed mild aortic regurgitation with an ascending aortic flap. The patient was started on labetalol and nitroglycerin to maintain systolic blood pressure below 120 mm Hg and was monitored for 30 minutes. As there was no extension of the dissection and the patient remained hemodynamically stable, a conservative approach was adopted, and further intervention was deemed unnecessary. A subsequent CT aortogram revealed proximal aortic dissection with a partially thrombosed false lumen (Figure 3, Video 2). At the 3-month follow-up, he remained stable with no residual flap or regurgitation on echocardiography; he was reluctant to undergo a follow-up aortogram because of financial concerns. This complication likely arose from aggressive postdilation, preventable with more conservative balloon sizing and inflation pressures.

## Patient 3

**Figure 4:**
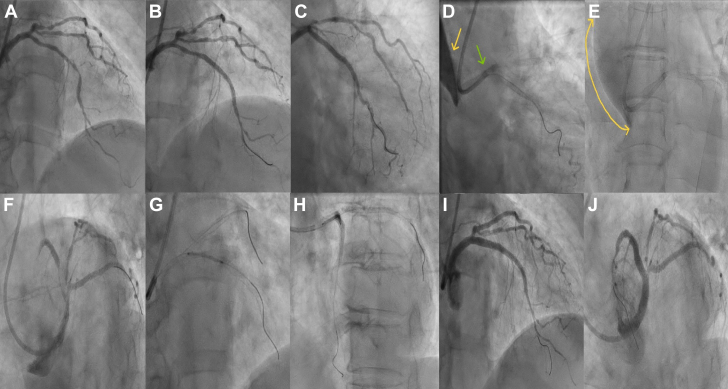
Angiographic Images of IACD Management From Patient 3 (A) Angiogram showing tight lesions in the LAD and LCX. (B and C) Good result after LAD and LCX stenting. (D) Dilation of the LCX stent resulted in type III IACD originating from the left main artery (yellow and green arrows). (E) Huge ascending aortic dissection seen during re-engagement (double-ended arrow). (F) Dissection propagation into the LAD with slow flow. (G and H) Stenting of the ostial LAD with extension into the left main artery. (I and J) Good final result with complete sealing of dissection. IACD = iatrogenic aortocoronary dissection; LAD = left anterior descending artery; LCX = left circumflex artery.

A 65-year-old woman with a recent inferior wall myocardial infarction underwent PCI to the left anterior descending artery (LAD) and left circumflex artery (LCX), 2 days after successful primary PCI to the RCA. The LAD was stented with 1 drug-eluting stent and the LCX was stented with 2 drug-eluting stents using a 6-F 3.0 extra-backup LAD catheter. During postdilation, catheter manipulation led to a Dunning type III IACD extending into the ascending aorta. As the patient remained hemodynamically stable, with no aortic regurgitation on echocardiography, we proceeded with stenting rather than surgical referral. Two additional drug-eluting stents were placed from the left main artery to the LAD and postdilated with a 4-mm noncompliant balloon to seal the dissection (Figure 4, Video 3). Serial echocardiography over the next 2 days confirmed the success of the intervention. She was doing well at the 2-month follow-up, but she declined a CT aortogram owing to financial constraints. This complication might have been avoided by gentler manipulation of the catheter.

## Patient 4

**Figure 5:**
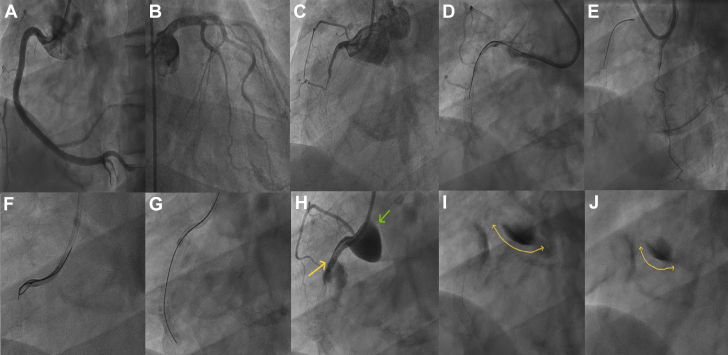
Angiographic Images From Patient 4 Illustrating RCA Stent Restenosis, Wire Entanglement, and IACD Development (A) Angiogram showing RCA poststenting in 2017. (B) Normal left-sided vessels in 2024. (C) Type IV in-stent restenosis of RCA stent in 2024. (D) Attempt to revascularize using Gaia-2 wire. (E) Gaia wire entanglement within the lesion. (F) Successful wire retrieval via balloon trapping. (G) Reattempt using another Gaia wire. (H) Angiogram revealing type II IACD originating from the RCA (yellow and green arrows). (I and J) Plane cine-angiogram showing gradual reduction in dissection after 10 and 20 minutes (double-ended arrows). Abbreviations as in Figure 2.

A 51-year-old man who underwent PCI to the RCA 6 years ago presented with 3 months of exertional dyspnea. Angiogram revealed an occluded RCA stent with normal left-sided coronaries. CTO recanalization began with 0.014-inch Fielder and Fielder XT guide wires (Asahi Intecc) using a Judkins right catheter. Subsequently, the approach shifted to an Amplatz left-1 catheter with a Gaia-2 wire (Asahi Intecc). During manipulation, the Gaia wire became entangled within the lesion. After several failed attempts to snare the wire, we deeply engaged the catheter and successfully extracted the entangled wire using balloon trapping. A second Gaia-2 wire crossed the proximal cap but failed to traverse the distal cap, leading to procedure abandonment. The final angiogram showed a Dunning type II IACD of the proximal RCA into the aorta (Figure 5, Video 4). Echocardiography revealed a dissection flap without aortic regurgitation. Given the absence of anterograde RCA flow, conservative management with close monitoring was chosen. Serial echocardiography over the next 2 days showed regression of the dissection flap. Cardiac magnetic resonance imaging performed after 6 weeks confirmed a sealed-off aortic dissection and nonviable RCA territory. The patient remained stable at the 6-month follow-up and was advised to continue conservative management. More careful wire handling and avoiding deep catheter engagement could have prevented this event.

## Discussion

Although iatrogenic complications such as IACD are avoidable by nature, effective prevention depends on a deep understanding of the predisposing factors and the procedural mechanisms. Dissections occur even with experienced operators, especially in complex and high-risk patients. The best strategy for managing IACD is to prevent its occurrence. Table 2 summarizes the predisposing factors associated with IACD.^4^^,^^6^ Coronary complexities and arterial challenges can complicate catheter engagement and increase injury risk, even with routine handling. Patient demographics matter: Older patients have fragile, calcified vessels, while female patients typically have smaller arteries, necessitating smaller, less stable catheters. However, the data on the association of demographic factors with IACD are inconsistent.^4^, ^5^, ^6^ Pre-existing cardiovascular risk factors and clinical context heighten these vulnerabilities, with acute coronary syndromes demanding rapid, aggressive approaches, whereas calcified plaques or CTOs require deliberate manipulation using specialized hardware and high-pressure dilations.^2^^,^^7^ Structural conditions can further weaken arterial walls, making them vulnerable to minor procedural forces. In our series, the sex distribution of the patients was equal, 2 of the patients were older than 55 years, and Dunning type III IACD was the most common variant.

**Table 2:** Predisposing Factors for Iatrogenic Aortocoronary Dissection

Category	Factors
Unfavorable coronary anatomy	• Ectopic or anomalous coronary artery origin • Shepherd's crook variant of RCA • Tortuous coronary arteries • Small ostia or proximal vessel diameter (<3.0 mm) • Atheromatous ostial lesion • Calcified coronaries • Acute coronary spasm
Challenging arterial access	• Tortuous or stenosed peripheral arteries (eg, iliac/subclavian) • High aortic origin of the innominate artery • Arteria lusoria • Brachial or subclavian loops • Radial artery spasm
Demographic risk factors	• Older age • Female sex
Clinical risk factors	• Traditional coronary risk factors (diabetes, hypertension, smoking) • Acute myocardial infarction • Chronic total occlusion
Genetic/Structural predisposition	• Genetic aortopathy (eg, Marfan syndrome) • Other structural weaknesses of the arterial wall

The mechanisms responsible for IACD include wedged contrast injections, deep catheter engagement trauma, subintimal guide wire entry, and aggressive coronary dilation, with wedged contrast injection being the most common.^6^^,^^8^ All of these mechanisms and predisposing factors, alone or in combination, can cause coronary artery dissection, which can extend forward (causing coronary occlusion), or backward into the aorta (causing acute regurgitation or branch occlusion), or, rarely, into the pericardium (causing tamponade). In our series, the IACD in patients 1, 3, and 4 stemmed from deep catheter engagement alongside wedged contrast injection, and the IACD in patient 2 was due to aggressive balloon dilation. The case of patient 4 highlights the risks of multiple manipulations after wire retrieval.

Early recognition and mitigation of dissection propagation are crucial in IACD management. Dissections can occur even in normal coronaries, and progression depends on contrast volume, injection force, and structural vulnerability. However, angiography often underestimates the extent owing to inadequate contrast. In our experience, for diseased or narrow ostia, the use of side-hole catheters reduces injection force and minimizes dissection risk, although the supporting data are limited.^4^^,^^6^ Intravascular ultrasound and/or fluoroscopic guidance are highly recommended over contrast modalities such as optical coherence tomography. We describe safe re-engagement strategies (the no-touch technique) in patients 1 and 3, as well as the use of intravascular ultrasound despite dissection in patient 1. This counters the traditional hesitancy around instrumentation postdissection, supporting cautious usage for interventional guidance. Noninvasive imaging includes echocardiography and CT angiography. Echocardiography is a cost-effective, readily available tool for assessing aortic valve integrity and dissection monitoring, ensuring no further progression and tracking new-onset valve insufficiency or hemopericardium. CT angiography confirms aortocoronary dissection and accurately evaluates the extent, guiding subsequent follow-up,^9^ but it is not essential if unaffordable. In our series, CT was performed in patients 1 and 2, and no follow-up scans were performed owing to financial constraints. All patients were monitored in the hospital for at least 2 days with echocardiography.

As per the literature, guiding catheters are more often associated with IACD compared with diagnostic catheters, with Amplatz left devices most commonly implicated, followed by Judkins catheters via transradial access; for right-sided coronaries, Judkins catheters are most commonly implicated, followed by Amplatz left through transfemoral approaches. Extra-backup catheters are more commonly associated with dissection of the left main artery.^6^^,^^8^ This may be partly attributed to their frequent use in complex interventions, in which inexperience or aggressive handling increases risk. Although nonconventional catheters are often implicated, the majority of IACDs in published reports involve commonly used catheters, likely owing to higher usage frequency.^6^ In our experience, the Judkins right catheter was the most frequently involved type in RCA-related dissections, and the extra-backup LAD catheter was responsible for left-sided interventions. Ultimately, no catheter is entirely safe, highlighting the importance of appropriately sized devices properly aligned with the artery. The RCA is more prone to IACD than the left coronary artery, given anatomical and size differences.^8^ The left main artery is generally larger and contains a higher proportion of smooth muscle cells and type I collagen fibers, contributing to its structural integrity and resistance to dissection.

IACD typically presents with chest pain and electrocardiogram abnormalities. About 25% of patients develop hemodynamic instability, sometimes requiring circulatory support.^4^ Arrhythmias occur in approximately 10% of cases, the most common being conduction defects necessitating a temporary pacemaker, followed by ventricular fibrillation/ventricular tachycardia. Although rare, cardiac arrest has been reported in roughly 2% of patients across various studies. In our series, patients 1 and 3 had electrocardiogram abnormalities without hemodynamic instability, resolving after ostial stenting. Patient 2 was managed conservatively with antihypertensive medications, and patient 4 remained asymptomatic.

**Figure 6:**
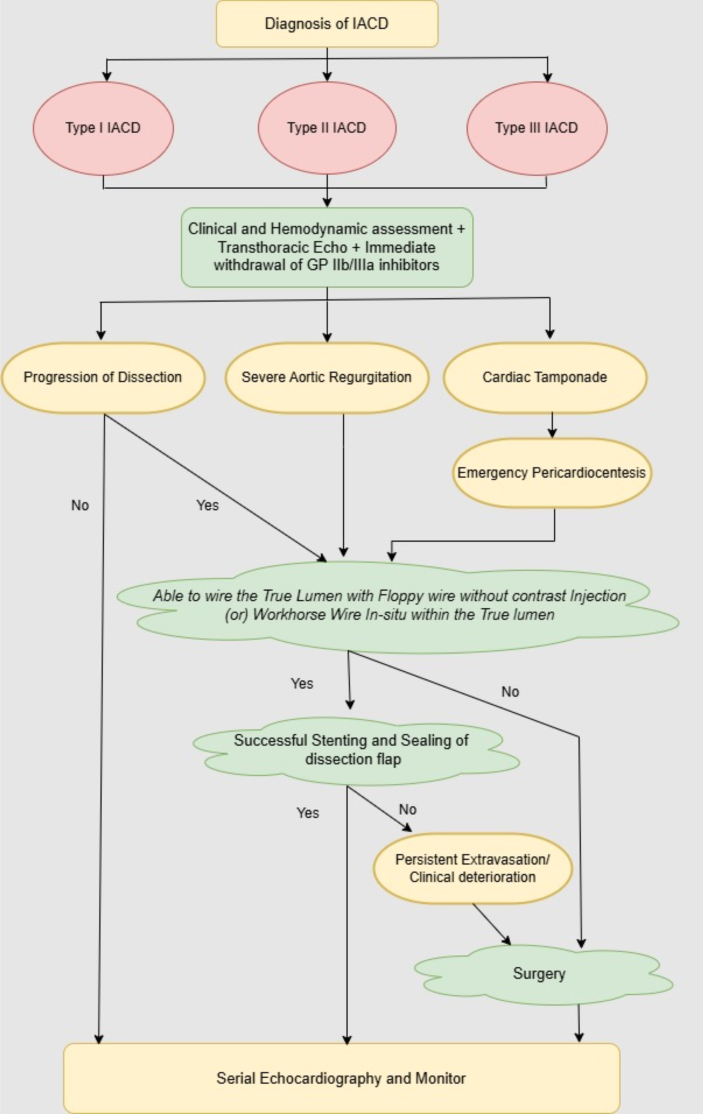
Flow Chart of IACD Management IACD management based on clinical stability, progression of dissection, and complications related to dissection. Conservative therapy is preferred for stable, localized cases, whereas stenting or surgery is indicated for unstable or extensive dissections. GP = glycoprotein; IACD = iatrogenic aortocoronary dissection.

Management strategies depend on clinical condition, hemodynamic stability, and progression of the dissection, rather than angiographic extent alone. Never panic during rare complications, even when the situation is unfamiliar. Multiple studies showed that stenting is the most common intervention (∼60%), followed by conservative management (∼30%) and surgery (<10%)^4^^,^^6^^,^^8^ (Figure 6). Ostial stenting using standard drug-eluting stents to seal the dissection entry point is standard practice. Covered stents are useful in perforations with extravasation.^10^ Nonetheless, the execution of emergency bailout stenting is challenging, with the risk of deterioration during the procedure. A conservative “wait and see” approach may be appropriate for stable patients with localized dissections and adequate coronary flow. We highlight the viability of conservative management even in angiographically extensive dissections, as in patients 2 and 4. However, this approach carries the risk of dissection progression, necessitating continuous monitoring.

Although surgery is rarely required, early cardiothoracic involvement is essential in extensive dissections or clinical deterioration. Surgical consultation should be considered in the presence of ≥1 of the following signs: persistent or worsening chest pain, hemodynamic instability, progression of dissection on imaging, severe aortic regurgitation, involvement of the aortic valve or arch, or cardiac tamponade. Emergency surgical repair is warranted when percutaneous sealing fails or dissection threatens vital aortic branches. In stable patients with progressive extension of dissection, delayed surgery may be planned. Decision-making should involve a heart team discussion, balancing procedural risks against surgical morbidity, especially in anticoagulated or post-PCI patients. In our series, ostial stenting was performed in 2 patients: one patient developed IACD poststenting and was managed conservatively, while the other patient was managed conservatively without stenting. Notably, no patients died, and all are doing well at follow-up.

## Conclusions

IACD, though rare, remains a serious complication of coronary interventions. Careful preprocedural assessment, thoughtful catheter selection, coaxial alignment, and gentle handling of catheters and wires are essential when multiple risk factors converge. Our case series emphasizes the need for constant vigilance during all coronary procedures. Once dissection occurs, minimize contrast use, consider intravascular ultrasound, and proceed with timely ostial stenting if needed. Conservative management may suffice in stable cases. Ultimately, prevention, vigilance, and technical precision are the keys to successful outcomes.

## Study Limitations

Among the study limitations, the retrospective design prevented the creation of a comparative group, limiting our ability to establish causation or evaluate management effectiveness. Planning a prospective study is challenging owing to the low incidence of IACD. In addition, this small case series may not represent the full spectrum of IACD, restricting its applicability to a larger population. Finally, the follow-up period varied among patients, limiting the assessment of long-term treatment outcomes.

**Visual Summary:**
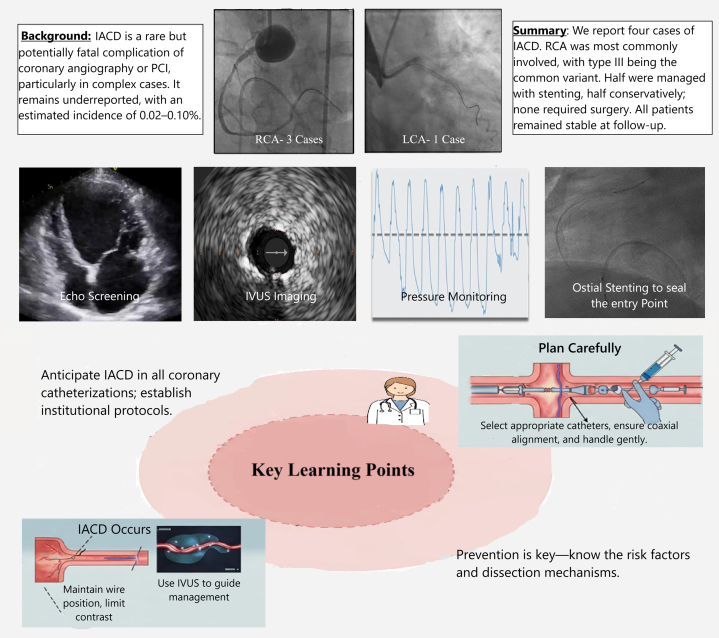
Iatrogenic Aortocoronary Dissection: A Brief Overview IACD = iatrogenic aortocoronary dissection; IVUS = intravascular ultrasound; LCA = left coronary artery; PCI = percutaneous coronary intervention; RCA = right coronary artery.

## Recommendations for the Future

A large-scale prospective study with extended follow-up will offer deeper insights into procedural effectiveness and optimize management strategies. Multicenter collaboration can enhance sample size and improve generalizability.

## Funding Support and Author Disclosures

The authors have reported that they have no relationships relevant to the contents of this paper to disclose.

## Appendix

Video 1 Angiographic and IVUS-Guided Ostial Stenting of IACD in Patient 1 The video demonstrates a recanalized mid-RCA lesion at presentation. Type III IACD was observed after predilation. There was an accidental disengagement of the guide wire; however, successful rewiring was achieved. An IVUS-guided stenting of the RCA was performed, extending up to the ostium. Unfortunately, there was incomplete coverage of the ostium, necessitating extension of the stenting. Final angiogram shows good flow, and computed tomography scan at discharge confirmed a sealed-off dissection. IACD = iatrogenic aortocoronary dissection; IVUS = intravascular ultrasound; RCA = right coronary artery.

Video 2 Conservative Management of Type III IACD After Ostial RCA Stenting in Patient 2 The angiographic video of patient 2 shows a significant lesion in the mid RCA at the time of presentation. After successful stenting, intravascular ultrasound assessment revealed the need for ostial extension of the stent. Postdilation of the proximal stent resulted in type III aortocoronary dissection. Conservative management with balloon occlusion stabilized the dissection. Postprocedural computed tomography confirmed a thrombosed false lumen with a patent stent. IACD = iatrogenic aortocoronary dissection; RCA = right coronary artery.

Video 3 Bailout Left Main Artery–LAD Stenting for Type III IACD in Patient 3 The video presents angiographic sequences from patient 3. Initially, the RCA was stented for a significant lesion. A few days later, angioplasty of the LAD and LCX was performed. After stenting the LAD and LCX, there was a distal edge dissection of the LCX stent. While addressing the distal edge dissection, an aortocoronary dissection occurred, leading to flow compromise in the LAD. Careful rewiring and stenting from the left main artery to the LAD successfully restored flow. IACD = iatrogenic aortocoronary dissection; LAD = left anterior descending artery; LCX = left circumflex artery.

Video 4 Aortocoronary Dissection After Failed RCA CTO Recanalization in Patient 4 The video presents angiographic footage from patient 4. It begins with a 2018 angiogram showing successful stenting of the RCA with a 4-mm stent. A follow-up angiogram 6 years later revealed CTO of the RCA stent. Multiple failed recanalization attempts led to guide wire entanglement, which was resolved using a balloon-trapping technique. A subsequent attempt resulted in aortocoronary dissection. Conservative management led to the gradual stabilization of the dissection. CTO = chronic total occlusion; RCA = right coronary artery.
